# Accurate diagnosis of lymphoma on whole-slide histopathology images using deep learning

**DOI:** 10.1038/s41746-020-0272-0

**Published:** 2020-05-01

**Authors:** Charlotte Syrykh, Arnaud Abreu, Nadia Amara, Aurore Siegfried, Véronique Maisongrosse, François X. Frenois, Laurent Martin, Cédric Rossi, Camille Laurent, Pierre Brousset

**Affiliations:** 1 Department of Pathology, University Cancer Institute of Toulouse-Oncopole, Toulouse, France; 2 Roche Institute, Boulogne-Billancourt, France; 3 ICube, University of Strasbourg, CNRS, Strasbourg, France; 4 Department of Pathology, Dijon University Hospital, Dijon, France; 5 INSERM UMR 1231, Dijon, France; 6 Department of Haematology, Dijon University Hospital, Dijon, France; 7 INSERM UMR 1037 Cancer Research Centre of Toulouse (CRCT), University of Toulouse III Paul-Sabatier, National Centre for Scientific Research (CNRS ERL 5294), Toulouse, France; 8 Laboratoire d’Excellence ‘TOUCAN’, Institut Carnot Lymphome CALYM, Toulouse, France; 9 Programme Hospitalo-Universitaire en Cancérologie CAPTOR (University Hospital Oncology Programme), Toulouse, France

**Keywords:** Haematological cancer, Machine learning

## Abstract

Histopathological diagnosis of lymphomas represents a challenge requiring either expertise or centralised review, and greatly depends on the technical process of tissue sections. Hence, we developed an innovative deep-learning framework, empowered with a certainty estimation level, designed for haematoxylin and eosin-stained slides analysis, with special focus on follicular lymphoma (FL) diagnosis. Whole-slide images of lymph nodes affected by FL or follicular hyperplasia were used for training, validating, and finally testing Bayesian neural networks (BNN). These BNN provide a diagnostic prediction coupled with an effective certainty estimation, and generate accurate diagnosis with an area under the curve reaching 0.99. Through its uncertainty estimation, our network is also able to detect unfamiliar data such as other small B cell lymphomas or technically heterogeneous cases from external centres. We demonstrate that machine-learning techniques are sensitive to the pre-processing of histopathology slides and require appropriate training to build universal tools to aid diagnosis.

## Introduction

The microscopic diagnosis of lymphoma remains challenging. In France through the Lymphopath network^1^, contrary to the UK or the US for example, we currently deal with a double interpretation rather than direct cases centralisation. Therefore, a diagnostic discrepancy is established when the referral pathologist sends the case for a second opinion with a proposal (with or without a signed report), which is unconfirmed by the expert^1^. Recent data from our group within the French (nationwide) Lymphopath network pointed out a 20% discrepancy between referral and expert pathologists directly impacting on patient care^1^. Other studies conducted in the US and the UK also demonstrated the impact of expert review on lymphoma management with variable rates of discordance (from 14.8 to 27.3%)^2–5^.

The diagnosis of lymphoma is currently based on histopathological examination of tissue sections at different magnification levels by a pathologist whose suspicion is based upon morphological features observed on haematoxylin and eosin (H&E) staining. Lymphoma diagnosis depends on the expertise of the pathologist who, in the case of follicular proliferation, has to clearly distinguish follicular lymphoma (FL) from follicular hyperplasia (FH), both being lesions that sometimes display very similar features. Finally, the ultimate decision relies on the use of immunostaining to reveal malignant germinal centres co-expressing Bcl2 and CD10^6^. However, approximately 10% of cases remain difficult (in particular those without expression of CD10 and/or Bcl2) and require additional tests such as fluorescent in-situ hybridisation or polymerase chain reaction techniques that are routinely unavailable in some laboratories.

Deep learning-based diagnostic systems have recently provided automated methods for histopathology image analysis^7–12^, which may reduce inter- and intra-observer variability in cancer diagnosis through an objective analysis^13^. Furthermore, digital microscopy allows the detection of characteristics previously unidentified by the human being and, combined with deep learning approaches, it could detect new morphological features^14,15^ and improve performance in histopathological interpretation. In a previous study^15^, the authors designed a computational pipeline to train a deep convolutional neural network (CNN) (inception v3)^16^ on whole-slide images (WSI) in order to recognise tumour versus normal lung tissue. This way they showed that deep learning models generated accurate diagnoses on lung histopathological images^15^. Similar deep learning approaches were also used in other recent studies to detect various cancer tissues, subtypes and related markers with highly reliable accuracy^8,11^. However, these highly-performant networks, applied to histopathological diagnosis, have all been designed without any method to control and quantify their uncertainty level. Moreover, the transition between benign and malignant tissues is not always a clear-cut issue and some images may be more difficult to classify than others. Information about the reliability of an automated diagnosis is essential in order this approach to be validated and integrated into diagnostic systems within medical practice, since the final decision must take into account the uncertainty level. This notion has already been addressed in recent work as a part of the automatic diagnosis of diabetic retinopathy from fundus images^17^. In the latter study, the authors showed the feasibility of associating deep learning-based predictions to uncertainty measures by using Bayesian approaches in order to improve diagnostic performance^17^.

In the field of haematopathology, the distinction between FL, the second most common subtype of lymphoma, and benign FH, is sometimes difficult to highlight on H&E-stained slides^1,6^. In the present study, we propose a deep learning-based framework to classify morphological changes occurring in FL or FH lymph nodes. For this purpose, H&E-stained WSI were used and processed at different levels of resolution (for a given pyramid level *n*, a full resolution image was downsized by a factor of 2^*n*^) with a stochastic Bayesian neural network (BNN). At this point, the aim of our work was not to provide a tool specifically dedicated to FL diagnosis but rather to explore BNN usefulness in order to classify images sharing numerous morphological features.

## Results

### A deep convolutional neural network enables an accurate automatic differential diagnosis between follicular lymphoma and follicular hyperplasia

**Fig. 1:**
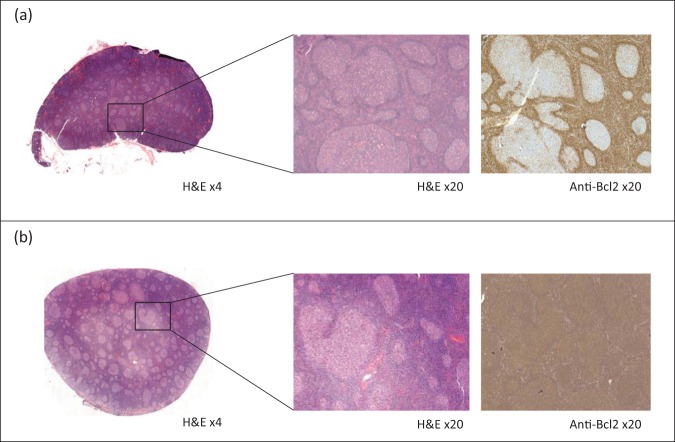
Histopathological distinction between follicular hyperplasia and follicular lymphoma (FL). The distinction between reactive hyperplastic germinal centres (**a**) and FL (**b**) can be difficult on haematoxylin and eosin (H&E)-stained slides making immunohistochemical stains mandatory for FL diagnosis: Bcl2 expression is down-regulated within reactive germinal centres (**a**) while it is expressed in the neoplastic cells in most FL cases (**b**).

We trained several deep CNNs to automatically distinguish FL from benign FH in lymph nodes on H&E digital slides. We used a total of 378 lymph nodes: 197 were infiltrated by FL (any grade, with or without Bcl2 expression, see Supplementary Table 1 for the clinical and histopathological characteristics) and 181 lymph nodes with FH. Representative WSI for each diagnosis are shown in Fig. 1. Networks were trained, validated and tested on independent datasets using 299 × 299 pixel patches obtained from the WSI at different resolution levels (i.e., numerical down-sampling of the image in micron/pixel), with 100–1000 patches per slide depending on the WSI size. A total of 320,000 patches were extracted, 160,000 of them were used for training (20,000 for each resolution level considered), 80,000 for validation sets and the remaining 80,000 for testing.

**Fig. 2:**
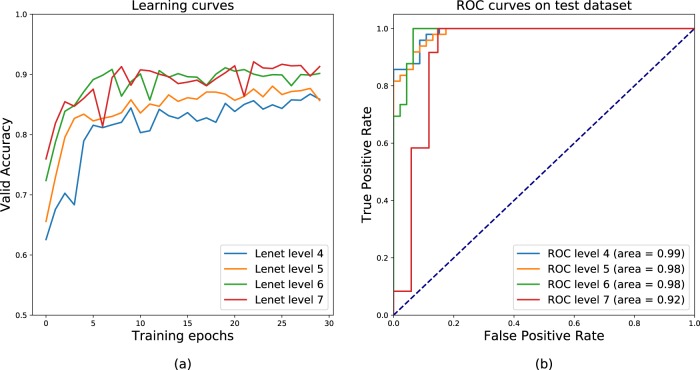
Learning and final ROC curves for slide diagnosis. **a** The model learns relevant information for patch classification as the patch level accuracy on the validation set increases with the number of learning iterations, at different resolution levels (7.84 µm/pixel to 62.72 µm/pixel). **b** Final ROC curves showing that the area under the curve (AUC) depends on the resolution level and could reach 0.99. Best slide diagnosis integration is obtained from patch classifiers with the smallest validation accuracy: at high resolution (pyramid level 4) more independent/non-overlapping patches are extracted and predicted, resulting in a more robust average prediction.

**Fig. 3:**
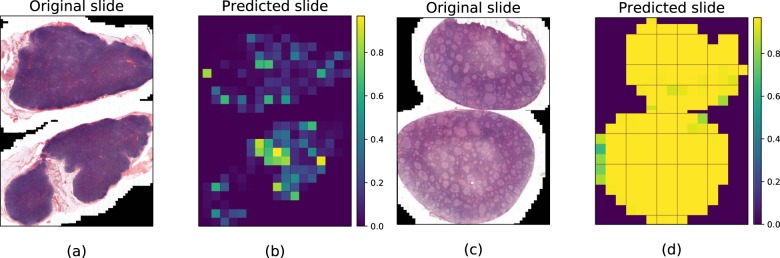
Patch classification visualisation on WSI for follicular hyperplasia (FH) and follicular lymphoma (FL) from the test set. Each predicted patch at resolution 7.84 µm/pixel is coloured by FL probability predicted by the system. **a** and **b** FH slide has very low FL probability on every patch. **c** and **d** FL probability is close to 1 everywhere on the FL slide.

These patches were analysed individually and patch-by-patch scoring was firstly performed by the networks. In the testing dataset, a total of 72,895 out of the 80,000 test patches were correctly diagnosed with the CNN model, yielding an overall 91% accuracy. Then, for a given slide and resolution level, diagnosis was performed by averaging patch predictions of the model trained at that level. This deep learning approach generated a diagnosis for each slide with an area under the curve (AUC) between 0.92 and 0.99 depending on the resolution level with a better diagnostic performance obtained at the lowest resolution level (Fig. 2). With optimum settings, acceptance of 20% of false alarms allowed 100% FL detection. Interestingly, best slide diagnosis integration was obtained for patch classifiers with the smallest validation accuracy (Fig. 2b): at high resolution (pyramid level 4), more distant patches were extracted and predicted, ending with a more robust average prediction (Fig. 2a). Visualisation of patch classification on WSI for FH (Fig. 3a) and FL (Fig. 3b) showed that FH slides had a very low FL probability on every patch, while FL probability was close to 1 everywhere on FL slides.

### Enhanced prediction performances by dropout variance ranking through a Bayesian neural network

**Fig. 4:**
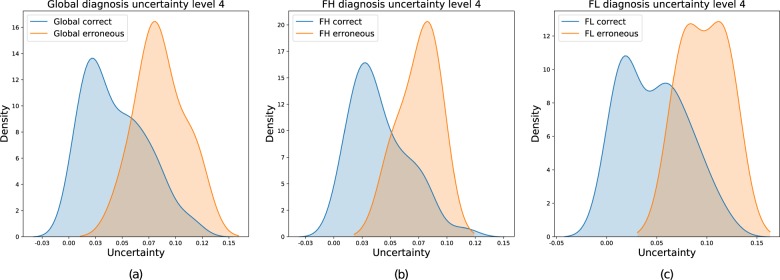
Erroneous predictions always correlate with higher uncertainty values. Uncertainty densities for any class patch prediction (FL or FH, i.e. global diagnosis) (**a**), FH prediction only (**b**), and FL prediction only (**c**). In each case, correct (blue) and erroneous (orange) diagnosis distributions are displayed.

Estimation of uncertainty, using a previously described BNN (see “Methods”) to predict FL or FH on our dataset, revealed that erroneous predictions always correlate with higher uncertainty values, regardless of diagnosis (Fig. 4).

**Fig. 5:**
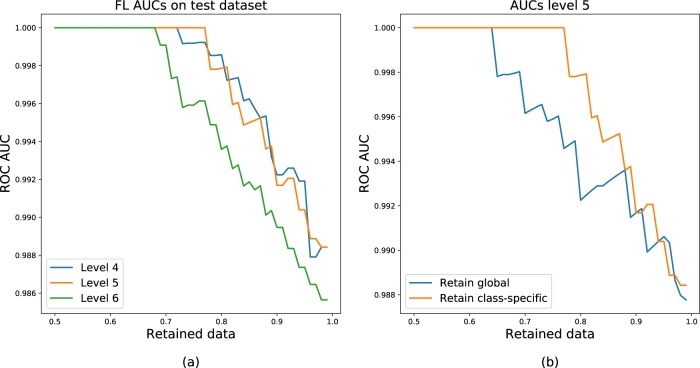
Improvement of Receiver-Operating Characteristics (ROC) via uncertain data removal. **a** ROC-Area under the curve (AUC) increases with uncertain data removal at different resolutions. **b** In the model trained at 15.68 µm/pixel (level 5), removing uncertain slides increases other slide diagnosis performance. Class-specific uncertainty thresholding (orange curve) appears to be superior to global uncertainty thresholding (blue curve) due to differences in FL/FH uncertainty distributions.

Dropout variance, a technique that randomly removes units from the network to sample the posterior distribution of the weights and approximate Bayesian inference^18^, appeared to be a powerful tool for increasing the system performance. For level pyramids 4 and 5, percentile of variance showed that removing 10% of the most uncertain slides led to perfect FL detection with around 2% false alarms and that AUC increased with high certainty thresholds (Fig. 5).

In the context of lymphoma screening, the aim is not to miss lymphoma diagnosis. Thus, it is worth noting that the system predicted FL (*σ* = 0.02) with more certainty than FH (*σ* = 0.04) and it made sense to have different certainty thresholds depending on the network output. Hence, class-specific thresholds for dropout variance led to a faster improvement in diagnosis, in reaching optimum accuracy by removing 23% of the most uncertain cases. With global thresholding, perfect accuracy was achieved only after removing 36% of cases (Fig. 5).

### Out-of-distribution inputs detection

Two types of out-of-distribution input (i.e. data different from those used in training and validating steps) were likely to alter the diagnostic performance and had to be considered: slides pre-processed in other centres (external data) subject to different staining protocols, and lymph nodes with lesions other than FL or FH (unfamiliar data).

**Fig. 6:**
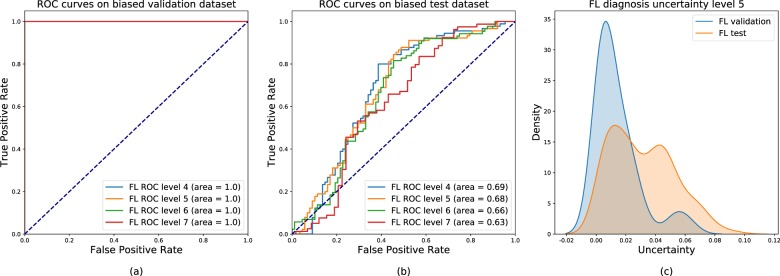
Receiver-Operating Characteristics (ROC) and uncertainty distribution for the biased dataset. **a** ROC curves on the biased validation dataset (composed exclusively by internal slides). **b** ROC curves on the biased test dataset (composed by internal and external slides). **c** Uncertainty distribution for validation data (blue curve) and test data (orange curve).

In order to evaluate the ability of our algorithm to assess data coming from other centres, we deliberately created a biased dataset with training/validation slides taken exclusively from our centre and a test set comprising external cases. After training, all the models showed perfect validation accuracies (AUC = 1.0) but were ineffective on external cases with an AUC falling between 0.63 and 0.69, depending on the resolution level (Fig. 6a, b). Nevertheless, a significant difference in uncertainty distributions appeared between internal and external data (Fig. 6c). In this experiment, setting *t* = 0.03 as a threshold on variance values led to the removal of no more than 10% of the internal data predictions, while it triggered the rejection of more than 50% of external data predictions. Yet, the uncertainty distribution of external cases appeared to be rather different compared to the ones of internal datasets and the break-up of unfamiliar data was at least partially possible by setting the appropriate threshold on the dropout variance. Thus, dropout variance in this case constituted an important statistical index of predictability of a set of cases by the algorithm.

**Fig. 7:**
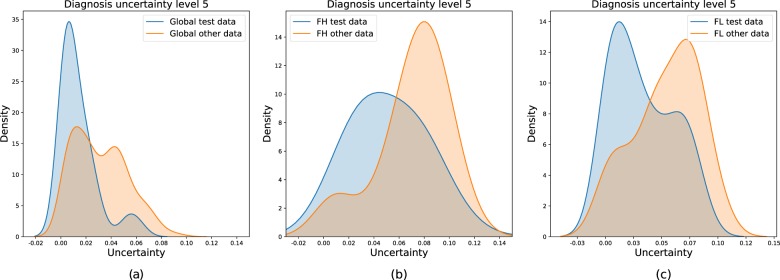
Comparison of uncertainty distributions between familiar test data (composed by FL and FH) and other unfamiliar data (non-FL/FH). Uncertainty distributions are shown for global (**a**) and class-specific (**b** and **c**) diagnosis. We separate test distribution (blue curves) and unfamiliar distribution (orange curves) on each plot. Non-FL/FH slides are diagnosed with higher values of uncertainty than the one encountered in usual FL/FH test data.

Other out-of-distribution inputs also comprised slides of lymph nodes affected by lesions other than FL or FH. To illustrate this purpose, we compared the uncertainty distributions between familiar FL/FH test data and unfamiliar non-FL/FH data for global (Fig. 7a) and class-specific (Fig. 7b, c) diagnosis. Non-FL/FH slides were still inherently diagnosed as FL or FH by the system, but decisions in that case correlated with higher values of uncertainty more likely than the one encountered with usual FL/FH test data. Hence, this finding proved the dropout variance ability to reject the out-of-scope dataset.

## Discussion

It is now widely admitted that most recent deep neural networks surpass humans in terms of visual recognition. Inter-observer variability amongst human beings is a very important issue, in particular for microscopic diagnosis with a direct impact on patient management. Several studies conducted in different countries have corroborated the impact of expert review on lymphoma management by comparing tumour diagnosis on referral with diagnosis of the same sample by an expert, and have reported variable discrepancy rates (from 14.8 to 27.3%) with an impact on patient care^1–5^. FL diagnosis might not be difficult in routine practice since many FL are readily suspected on H&E slides. Nevertheless, as shown in Fig. 1, histopathological distinction between FL and benign FH can be challenging and the absence of robust and definitive morphological features on conventional optical microscopy makes necessary the use of immunohistochemical staining in order to confirm diagnosis^6^. However, difficult FL cases may be overlooked if the pathologist does not suspect lymphoma and does not perform complementary immunohistochemical analyses. In addition, 10% of cases are Bcl2 negative and entail diagnostic issues. Providing an automated and exhaustive WSI analysis and exploring characteristics that cannot be identified by a pathologist on conventional optical microscopy, deep learning approaches add a new dimension to morphological analysis. Herein we have demonstrated the effectiveness of deep learning in the diagnosis of FL on H&E digital slides, including Bcl2-negative FL that are often difficult to diagnose despite the use of immunohistochemistry. Noting that malignant tissues have both cellular and structural atypia and thus different magnifications would each confer important information, WSI were processed at multiple resolution levels.

Another innovative approach has been the use of BNN, providing a level of uncertainty regarding output data. In our model, and as previously observed by Leibig et al.^17^, incorrect predictions of the algorithm on familiar data were characterised by higher variance values (i.e. uncertainty level), and ROC AUC increased for all networks as uncertainty tolerance decreased. On the one hand, the effectiveness of this approach confirms our model validity. On the other hand, this implementation of certainty in our network enables the identification of cases that had not been used for training (external cases or other diagnosis) in order to disregard predictions on these cases. For example, our BNN has been trained to separate FL from FH but its uncertainty level did not allow any consideration of the diagnoses proposed on slides of lymph nodes affected by other subtypes of small B-cell lymphomas (important decreases in AUC and certainty levels).

The visualisation of patch classification on WSI showed that FH slides had very low FL probability on every patch whereas FH probability was very low everywhere on FL slides. Thus, it is tempting to speculate that the algorithm should also be effective on smaller samples such as needle biopsies, for which immuno-morphological interpretation may be very challenging, even for an expert pathologist.

By building a biased dataset, we also confirmed that these machine-learning techniques were very sensitive to the pre-processing steps. Despite their evolution out from Bayesian frameworks^18,19^, neural networks abide by the laws of statistics and require a representative sample to achieve coherent inductive reasoning. Therefore, efforts to adapt the system to different staining protocols or different types of scanners are facilitated by training on multi-centre datasets. However, universal applicability is never guaranteed. As an extreme example, our biased dataset experiment showed that, despite relative differences in their certainty distribution, case-by-case separation of external test data from internal data remained impossible considering dropout variance only. Indeed, removing 10% of the most uncertain internal slides (in the validation set) fixed a threshold on variance values which led to the rejection of 50% of external test cases. Moreover, the accuracy on the remaining 50% of the most certain predictions was as low as on the entire test set (between 63 and 69%), showing that certainty did not even correlate with prediction accuracy on external cases. Yet, dropout variance still stood as a relevant index of predictability since the 10–50% ratio was statistically favourable. Given that total removal is impossible and that, in any deployment scenario, it only acts as a temporary solution, dropout variance may be used in a pre-production phase to automatically collate uncertain slides that the system has not been properly trained to evaluate. Thus, these uncertain slides could be collected to build a complementary dataset that may eventually be used to compile a new training stage to fit with data from new centres. The most relevant issue in this kind of approach is the quality and homogeneity of input data. Indeed, the best accuracy was reached with training, validation and test sets of slides homogeneously processed in the same pathology department. In those particular conditions, maximum accuracy was achieved with a limited number of training cases. However, when the slides used for testing were processed in laboratories other than training ones, the accuracy dropped dramatically. This drawback, which has also been recently reported by Jones et al.^20^, is easy to understand but difficult to explain as CNNs represent black boxes in which decisions are made on features automatically extracted by networks. In other words, CNNs are able to precisely detect and integrate slide technical variability in their decision making process. In our study, we clearly show that this problem could be solved when training and validation sets were composed of slides from multiple origins. This strongly suggests that the development of robust artificial intelligence solutions helping pathologists in their diagnostic decision making should rely on large training sets with diverse microscopic features and heterogeneous technical processing.

Our approach is really unique and innovative since it considers tissue section/image processing heterogeneity as a major drawback in the design of robust diagnostic assistance tools based on deep learning. Indeed, with the advance of digital pathology, various machine learning models have been developed to try to automate histological images analysis. While accuracy is optimised by most of these algorithms, few of them provide the reliability level of their predictions, a feature we deem essential in order to consider the deployment of these tools in medical practice. Bayesian approaches have recently been successfully used for whole-slide segmentation^21^, but to our knowledge, they have never been employed to diagnose malignancy on WSI. Another major novelty in our approach is to weight the predictions with a level of certainty. We also show that uncertainty measurements can be used to detect “outsider” images that should not be interpreted, whether it is a different pathology or FL/FH samples with image heterogeneity due to different technical procedures. Our findings need to be strengthened over multiple independent cases cohorts. Nevertheless, they suggest that this innovative deep-learning model, that combines extracted data at different resolution levels, may be a promising tool for lymphoma screening in the age of digital pathology, after an appropriate learning process. In the French Lymphopath network, we have collected more than 100,000 lymphoma cases encompassing all the clinical-pathological entities listed in the WHO classification^22^ from multiple sources. This dataset represents unique material that could be used to provide a robust supportive framework for lymphoma diagnosis in routine practice and this approach should also be used in diagnosing other pathologies on H&E digital slides.

## Methods

### Image datasets

To develop and evaluate our diagnostic algorithm, we retrospectively collected a total of 378 H&E-stained WSI of lymph nodes with a diagnosis of FL (*n* = 197) or FH (*n* = 181). All FL cases were retrieved from the lymphopath database in two pathology departments (Toulouse University Cancer Institute and Dijon University Hospital, France) and were previously investigated by immunohistochemistry. The FH cases, including reactive lymph nodes with few hyperplastic reactive germinal centres to florid FH, were collected in the pathology department of Toulouse University Cancer Institute, France. In another step, in order to assess our system ability to detect out-of-distribution inputs, we also included 65 H&E-stained WSI of lymph nodes affected by other small B cell lymphomas (referred to in the text as unfamiliar non-FL/FH) such as chronic lymphocytic leukaemia/lymphoma, mantle cell and marginal zone lymphomas diagnosed at the University Cancer Institute of Toulouse.

Tissue samples were collected and processed at the “CRB Cancer des Hôpitaux de Toulouse”, following ethical procedures (Declaration of Helsinki) and after obtaining written informed consent from patients. In accordance with French law, the “CRB Cancer des Hôpitaux de Toulouse” Cancer collection was reported to the Ministry of Higher Education and Research (DC 2009–989). A transfer agreement (AC-2008-820) was obtained following approval by the ethics committees. Similarly, CRB Cancer du CHU de Dijon (University Hospital Centre) data has been reported to the Ministry of Higher Education and Research (DC-2008-508) and a transfer agreement (AC-2008-750) was granted too. Clinical and biological annotations of the samples have been declared to the “Comité National Informatique et Libertés” (CNIL—French Data Protection Authority). The study was approved by the local ethical committee (Comité de Protection des Personnes Sud-Ouest et Outremer II). After anonymization, the H&E-stained slides were digitised with a Panoramic 250 Flash II digital microscope (3DHISTECH, Budapest, Hungary) equipped with a Zeiss Plan-Apochromat 20× NA 0.8 objective and a CIS VCC-FC60FR19CL 4 megapixel CMOS sensor (unit cell size 5.5 × 5.5) mounted on a 1.6× optical adaptor, to achieve a scan resolution of 0.24 µm^2^/pixel in the final image.

In the first experiments, all H&E WSI of FL and FH (from the pathology departments of the University Cancer Institute of Toulouse and University Hospital of Dijon, France) were randomly divided into training (50%), validating (25%), and testing (25%) datasets. For each WSI, we sampled 299 × 299 pixel image patches over a regular grid, removing the patches covered with less than 50% tissue. Unaware of the amount of context and spatial resolution required by networks to recognise FL and FH patterns, all patch images were extracted at eight resolution levels (i.e. numerical image down sampling) ranging from 0.49 to 125.44 µm/pixel, enabling model fitting and evaluation over these resolutions for later selection or combined predictions.

Machine-learning algorithms can be very sensitive to multi-centre deployment. In a final experiment and for illustrative purposes, we deliberately constructed a biased dataset with training and validation sets, each containing slides exclusively picked from our centre (internal cases). The biased test set comprised a mixture of other internal (i.e. from the University Cancer Institute of Toulouse, *n* = 24) and external (i.e. from the University Hospital of Dijon, *n* = 24) FL cases. This experiment enabled us to assess the diagnostic performance of our network on samples that the system has not been properly trained to predict.

### From patch prediction to whole-slide diagnosis integration

Following the probabilistic formalism of diagnosis, trained deep-learning classifiers are usually considered as final decision makers given their ability to produce accurate class predictions from images^23–25^. As the computational cost of forwarding WSI at sufficient resolution to a CNN remains prohibitive, we therefore placed our work in the patch-based classification framework. This approach has already been extensively explored in the domain of remote sensing and recently implemented with success in digital pathology^15^. We thus proposed to train deep CNN architectures to predict the presence of FL/FH patterns in non-overlapping tiles of WSI. All of the architectures studied had a final layer with 2 softmax activated units coding for the probable presence of FL and FH. Final tile labelling was allocated by the unit with maximum output.

Datasets were built with the objective of minimal slide annotation, without manual selection of slide regions by a pathologist. In this respect, in the training dataset, all patches extracted in an FL slide were labelled as “FL”, and conversely, “FH” label was given to all patches extracted from a FH WSI. According to common histopathological notions, we assumed that, when present, FL pattern involved almost all the specimen surface, and that the system did not need any region delineation to perform its classification.

WSI patch classification generally leads to semantic segmentation where the most represented label is likely to be the correct disease. However, considering the spatial constancy of a given pattern in a WSI, CNN predictions of non-overlapping tiles can also be seen as multiple independent measurements statistically centred on the correct diagnosis. Intuitively, and in accordance with coherent Bayesian inference, the average measurement appears to be a favourable estimation of global WSI diagnosis, leading to an increase in confidence related to the number of observations (i.e. number of predicted patches). Consequently, with our CNN, each WSI classification was performed by averaging individual patch predictions over the whole slide.

### Risk assessment

Effective uncertainty estimation from deep neural network models is an active topic of research and appears to be a key-concept for their settlement in practical healthcare systems. One way to provide certainty information from a deep learning model is to use a BNN that models the system weights as random variables with densities conditioned by the training data^19^. The model is said to be “confident” about its predictions if they remain consistent while sampling the weights distribution. However, the evaluation of the posterior distribution of weights, even for simplistic priors (typically Gaussian), remains computationally expensive and BNN properties are therefore obtained by approximations. We implemented the approach developed by Gal and Ghahramani^18^ who proposed the use of dropout, a technique that randomly removes units from the network, to sample the posterior distribution of the weights and approximate Bayesian inference. Despite being controversial for using the term “uncertainty” with the incompatible “Bayesian uncertainty” definition^24^, this method has proved to be practically useful for automating medical decisions with almost no implementation or computation time overheads^16^. Thus, for a single input image, multiple forward passes were performed with different dropout draws. Average value (*µ*) and variance (*σ*) over the multiple dropout predictions were respectively taken as final decision and uncertainty. The notion of uncertainty can be split up into two practical requirements; one being its ability to estimate the difficulty of classifying an input belonging to its application scope, which is related to the network calibration and relates prediction values to its true accuracy^26^. The other consists in predicting whether an input image is close enough to the training data to ensure reliable classification by the system. In our study case, dropout network prediction variance handles both specifications, which seem to support its utility in the field of automatically assisted medical decisions. The plots for all uncertainty distributions were obtained by probability density approximation from histograms (Gaussian kernel fitting) provided by the Seaborn Python package.

### Reporting summary

Further information on experimental design is available in the Nature Research Reporting Summary linked to this article.

## Supplementary information

Supplementary Information

Reporting Summary

## Data Availability

All relevant data that support this study findings are available from the corresponding author upon reasonable request.

## Code availability

A complete Python implementation of the experiments described in this article is available on a public repository: https://github.com/ArnaudAbreu/DiagFLFH.
