# Supplementary material for: Accurate diagnosis of lymphoma on whole-slide histopathology images using deep learning
Source: NPJ Digit Med. 2020 May 1;3:63. doi: 10.1038/s41746-020-0272-0 (PMC7195401; doi:10.1038/s41746-020-0272-0)
Supplement: Supplementary file 1 — Supplementary Information [file 41746_2020_272_MOESM1_ESM.pdf]

Variable	No (%)
**Median Age (range)**	62y (29-88)
**Sex**	
Male	111 (57.8 %)
Female	81 (42.2%)
**Bcl2 (immunohistochemistry)**	
Positive	186 (94.4%)
Negative	11 (5.6%)
**Grade**	
1-2	180 (91.4%)
3A	14 (7.1%)
3B	3 (1.5%)
**Original department of pathology**	
University Cancer Institute of Toulouse, France	173 (87.8%)
University Hospital of Dijon, France	24 (12.2%)

Supplementary Table 1: Clinical and histopathological characteristics of the 197 follicular lymphoma cases used in the study.
